# Sildenafil effect on testosterone-induced prostate hypertrophy and relaxation of urinary bladder neck muscles

**DOI:** 10.1016/j.toxrep.2025.102080

**Published:** 2025-06-30

**Authors:** Qasim A. El-Dwairi, Karem H. Alzoubi, Rania Mahafdeh

**Affiliations:** a Department of Anatomy, Faculty of Medicine, Jordan University of Science & Technology, Irbid 22110, Jordan; b Department of Clinical Pharmacy, Faculty of Pharmacy, Jordan University of Science and Technology, Irbid, Jordan; c Department of Doctor of Pharmacy, Faculty of Pharmacy, Jadara University, P.O. Box 733, Irbid 21110, Jordan

**Keywords:** Sildenafil, Testosterone, Prostate Hypertrophy, Urinary Bladder, Neck Muscles

## Abstract

**Background:**

Previous studies have demonstrated the expression of phosphodiesterase-5 receptors in prostate tissue. In this study, we investigated the efficacy of sildenafil citrate in testosterone-induced benign prostate hyperplasia (BPH) in rabbits.

**Methods:**

Prostate hyperplasia was induced using testosterone propionate for 8 weeks. Rabbits were divided into two groups: control and experimental. The experimental group was further subdivided into two subgroups: one subgroup was sacrificed after testosterone induction, while the other subgroup received sildenafil (5 mg/kg/day) via intragastric intubation for 8 weeks. The weight of the prostate and relaxation of the bladder neck muscle were assessed. Organ bath experiments evaluated the effect of sildenafil on phenylephrine-precontracted bladder neck muscle strips.

**Results:**

The mean prostate weight was reduced by 65.34 % after 8 weeks of treatment with sildenafil in animals with BPH. Sildenafil induced significant smooth muscle relaxation of the phenylephrine-contracted bladder neck muscle strips. The mean relaxation value was 2.11 ± 0.13, representing a 32.8 % reduction in contraction percentage. Maximal relaxation was produced at 5.0 × 10⁻⁶ M of sildenafil. Sildenafil induced significant relaxation of the phenylephrine-contracted bladder neck muscle strips. Adding the nitric oxide synthase inhibitor L-NAME inhibited relaxation, whereas sodium nitroprusside, a nitric oxide donor, increased it. Histopathological analysis showed increased papillary projections, acinar areas, and epithelial thickness in the testosterone-treated group. Sildenafil treatment reversed the hypertrophic and hyperplastic changes.

**Conclusions:**

The results indicate that sildenafil may provide a dual function in the treatment of erectile dysfunction and the relief of urinary tract complications associated with prostatic hypertrophy.

## Highlights

• The efficacy of sildenafil citrate in testosterone-induced BPH in rabbits was investigated.
• Sildenafil reduced prostate weight and reversed histological changes associated with hyperplasia.
• Sildenafil induced relaxation of bladder neck smooth muscle strips in testosterone-treated rabbits.

## Graphical Abstract

**Figure:**
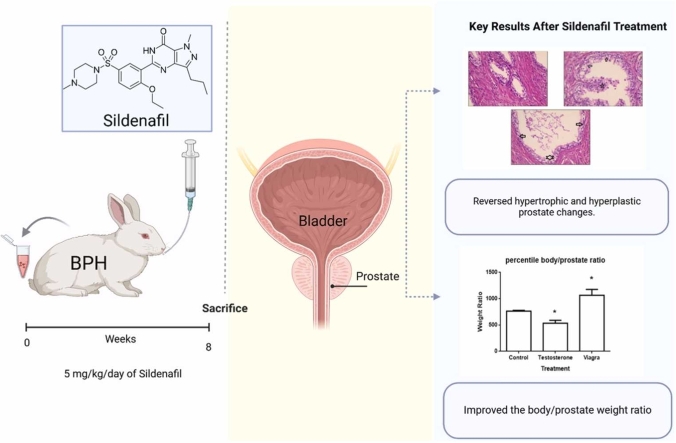

## 1 Introduction

Benign prostate hypertrophy (BPH) is a widespread disorder affecting over 70–80 % of aged men and resulting in lower urinary tract disturbances such as lower urinary tract symptoms (LUTS) and erectile dysfunction (ED) [1]. The prevalence of BPH increases with age [2]. According to autopsy studies, the age-specific prevalence of BPH has been estimated to be 8 % in the fourth decade of life, 50 % in the sixth, and 80 % in the ninth [3], [4]. Compensatory bladder hypertrophy causes irritants, such as obstructive symptoms, because of reduced urinary flow. The condition may progress to bladder dysfunction as an end stage of this disease, increasing the risk of urinary tract infections and renal failure. Therefore, preventing this progression is strongly recommended [5], [6].

The potential impact of pharmacologic targets on the pathophysiology of urination remains poorly understood. Several studies have indicated that drugs such as α_1_ adrenoreceptor antagonists (such as Terazosin and Doxazosin) [7] and 5α-reductase inhibitors (such as Finasteride) [8] may reverse such changes in the prostate of elderly men with significant improvement in the treatment of erectile dysfunction [5], [6]. However, both treatments have known limitations and side effects, underscoring the need for new therapies targeting distinct pharmacological pathways that can offer more acceptable BPH treatment options for patients [9].

Sildenafil (Viagra®) is an oral phosphodiesterase inhibitor type 5 (PDE5Is). Since it obtained FDA approval in April 1998, Sildenafil has been a well-tolerated oral medication widely used in the treatment of male erectile dysfunction [10]. Sildenafil enhances the relaxation of urogenital smooth muscle in the corpus cavernosum during sexual stimulation via modulating the nitric oxide/cyclic guanosine monophosphate (NO/cGMP) cascade [10]. Activation of non-adrenergic, non-cholinergic neurons in the genitourinary system promotes nitric oxide synthase (NOS), the enzyme that triggers NO release from the terminal nerves. Then, NO enters the surrounding tissues and activates the enzyme guanylyl cyclase (GC), which triggers the conversion of guanosine triphosphate (GTP) to the second messenger cGMP. cGMP leads to lower calcium levels, relaxes smooth muscle, and increases blood flow, leading to penile erection. cGMP is mainly metabolized by cGMP type 5 phosphodiesterase type 5. So, using PDE5Is prevents cGMP breakdown by inhibiting phosphodiesterase (PDE), sustaining cGMP levels, and supporting erectile function [6], [10]. Randomized placebo-controlled studies hypothesized that impaired NO/cGMP pathways contribute to prostate pathophysiology [11], [12]. PDE5Is have demonstrated efficacy in treating ED and LUTS by modulating the NO/cGMP pathway, with a favorable safety profile compared to α_1_ adrenoreceptor antagonists [11], [12].

The association of LUTS with erectile disease is well established, and studies recently demonstrated a benefit in LUTS with sildenafil treatment [13]. Several investigators have attempted to create an animal experimental model of BPH to mimic the conditions that occur in humans. BPH has been induced in mice [14], Rats [15], and dogs [16]. However, in our study, we chose the rabbit model to evaluate the relaxant effect of sildenafil on the urogenital tract. The rabbit prostate gland demonstrated soft tissue density like that of humans, and the rabbit model also provides sufficient smooth muscle tissue for evaluating sildenafil's relaxation effects [17]. We aimed to determine the pharmacological basis for sildenafil's application in BPH treatment. We specifically evaluated the relaxant effects of sildenafil on bladder neck smooth muscle and assessed its potential to reverse testosterone-induced prostate hypertrophy.

## 2 Methods

### 2.1 Animal model and experimental design

Thirty-adult male New Zealand white rabbits (24–32 weeks old) were used. The rabbits were purchased and housed in the animal house at Jordan University of Science & Technology (JUST). They were fed pellets and carrots ad libitum and housed in accordance with the institutional guidelines and regulations for animal care. The Institutional Animal Care and Use Committee of JUST (IACUC-JUST) approved the study protocol (Protocol number 20060134). Additionally, the study adhered to the internationally accepted standards of animal research following the 3Rs principle (Replacement, Reduction, and Refinement).

The rabbits were randomly divided into three groups: Group 1 (Control, n = 10) received no treatment (vehicle only). Group 2 (Testosterone-induced hyperplasia, n = 20): subcutaneously received testosterone propionate (7 mg/kg/day, Sigma-Aldrich, St. Louis, MO, USA) for 8 weeks to induce prostate hypertrophy. Following 8 weeks, Group 2 was further divided into two subgroups: Group 2 A (n = 10): Rabbits were sacrificed, and the prostates and bladder necks were dissected. Prostate weights were recorded and compared to Group 1 (Control group). Bladder neck muscle strips were prepared for organ bath experiments. Group 2B (n = 10): Received sildenafil (5 mg/kg/day, Sigma-Aldrich, St. Louis, MO, USA) via intragastric intubation for 8 weeks, a duration that corresponds to one complete spermatogenic cycle in rabbits, independent of the animals' initial age. After treatment, all rabbits survived until the scheduled sacrifice day. Rabbits were anesthetized in the induction chamber using 5 % isoflurane in oxygen and continued until respiratory arrest occurred; this was followed by decapitation to ensure euthanasia, as previously described [18], [19]. Then, the prostate weights were recorded in comparison with Group 2 A.

### 2.2 Organ bath experiment

Bladder neck muscle strips (0.5 ×0.3 cm) from control and testosterone-treated rabbits were dissected under a microscope. The strips were placed in an organ bath filled with Krebs solution containing the following concentrations (mmol/L): NaCl, 115; KCl, 4.6; MgSO4, 1.2; CaCl2, 2.5; NaHCO3, 25; KH2PO4, 1.2; glucose, 11.1; and disodium ethylenediaminetetraacetic acid, 0.01. The solution was equilibrated with 95 % oxygen and 5 % carbon dioxide to achieve a pH of 7.3–7.4 and was then kept at 37°C. One end of the strip was attached to the organ bath wall, while the other was connected to a strain gauge to measure tension (Model Grass FT03); Grass Instrument Division of Astro-med. Inc. West Warwick, RI).

After stabilization, the strips were pre-contracted with phenylephrine (10^-6 M). Sildenafil-induced relaxation was assessed by constructing cumulative concentration-relaxation curves. Relaxation responses were also tested in the presence of N^w^-Nitro-L-Arginine methyl ester (L-Name, 10^-4 M, Sigma-Aldrich, St. Louis, MO, USA), a nitric oxide synthase inhibitor, or with Sodium Nitroprusside (SNP, Sigma-Aldrich, St. Louis, MO, USA), a nitric oxide donor, respectively.

### 2.3 Histology study

Prostates of all groups were dissected out immediately following euthanasia at the end of their respective treatment periods. Group 2 A rabbits were sacrificed after 8 weeks of testosterone administration, while Group 2B rabbits were euthanized after completing the additional 8 weeks of sildenafil treatment. The tissues of all groups were then dissected out, cut into 2–3 mm blocks, fixed in 10 % neutral buffered formalin overnight, and then washed under tap water for 2 h. Prostate specimens were then dehydrated with gradual ethanol concentrations and cleared with Xylene. Sections were embedded in paraffin blocks at room temperature. Five-μm paraffin sections of these blocks were stained with hematoxylin and eosin (H&E) for microscopic examination and histopathological analysis. The number of papillary projections per acini, acini number, acinar area, and interstitial areas were determined. Furthermore, the epithelial thickness was measured, and all results were tabulated and compared to the control and testosterone-treated groups.

### 2.4 Statistical analysis

Data were represented as mean ± Standard Deviation (SD). GraphPad Prism (version 4.0, GraphPad Software, La Jolla, CA) was used to perform all statistical analyses. A one-way ANOVA followed by Tukey’s post-hoc test was used to compare the three groups. A paired *t*-test was used to compare the two groups (Sildenafil-induced relaxation in bladder neck smooth muscle). P < 0.05 was considered statistically significant.

## 3 Results

### 3.1 Impact of sildenafil on the measurements of prostate weight

Administration of testosterone propionate for 8 weeks resulted in prostate hyperplasia in rabbits. The mean prostate weight for the control group was 835.22 ± 8.18 mg (± SD). In contrast, the rabbits with prostate-induced hyperplasia had a significantly higher mean prostate weight of 1375.30 ± 19.15 mg (±SD). The treatment of this group with sildenafil for 8 weeks reduced the prostate weight to 900.10 ± 45.51 mg (± SD). This reflects a significant (p < 0.001) decrease in prostate weight by 34.56 %, indicating that sildenafil may effectively reverse the cellular changes associated with prostate hypertrophy.

### 3.2 Effect of sildenafil on body/prostate weight ratio

**Fig. 1:**
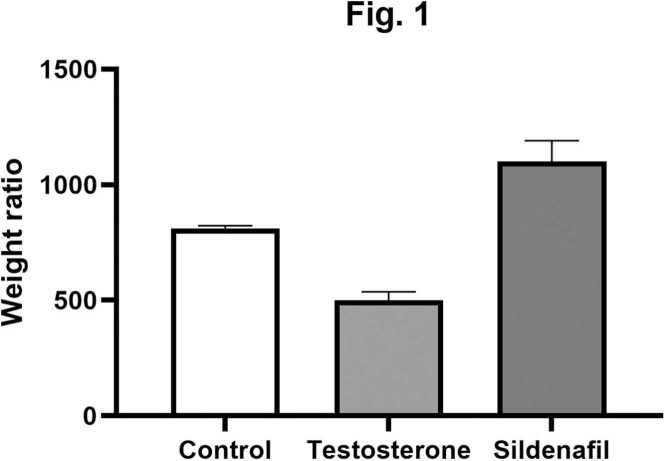
Body/prostate weight ratio in control, testosterone-induced hyperplasia, and sildenafil-treated groups. Asterisks (*) indicate statistically significant (p < 0.05) compared to control groups.

Fig. 1 illustrates the body/prostate weight ratio across the three groups. The control group showed a mean ratio of 811.54 ± 12.2. Testosterone treatment significantly reduced this ratio to 501.21 ± 35.10 (p < 0.001), confirming the induction of prostate hypertrophy. In contrast, sildenafil treatment increased the ratio to 1100.51 ± 89.90, which was significantly higher than both the testosterone and control groups (p < 0.001). These findings indicate that sildenafil not only reversed testosterone-induced changes but also improved the body/prostate weight ratio beyond baseline levels. This marked increase suggests a reversal of prostate enlargement relative to overall body mass. The elevated ratio following sildenafil treatment implies a therapeutic benefit in reducing hyperplastic prostate growth, restoring structural and physiological homeostasis.

### 3.3 Histological analysis of prostate tissue

**Fig. 2:**
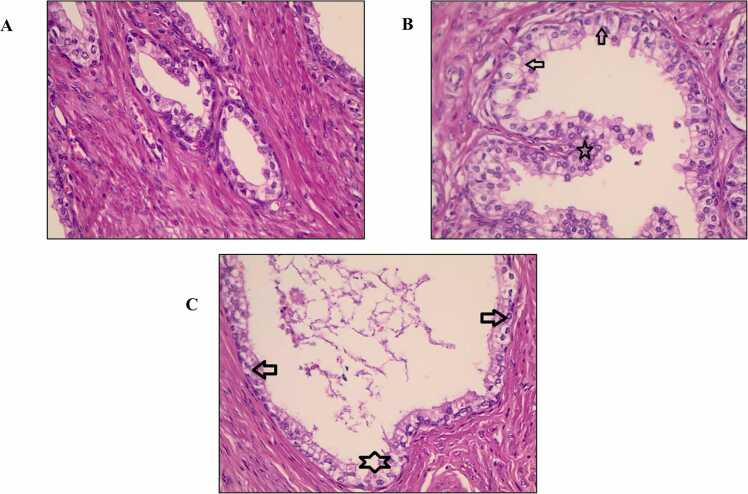
Histological analysis of prostate tissue from different experimental groups: (A) Control group (100X), (B) testosterone-treated group (400X), and (C) Sildenafil-treated group (400X).

As shown in Fig. 2**A–C**, the histological examination of prostate tissue revealed distinct structural differences among the control, testosterone-treated, and sildenafil-treated groups. The prostate sections of the control group exhibited typical structures of acini and epithelium without papillary projections, indicating healthy prostate tissue **(**Fig. 2**/A)**. In contrast, the testosterone-treated group resulted in marked histological changes consistent with prostate hyperplasia **(**Fig. 2**/B)**. These changes included a significant increase in papillary projection, a marked increase in acinar area, and increased lining epithelium thickness compared with the control and sildenafil-treated groups. The sildenafil-treated group ameliorates testosterone-induced hypertrophic/hyperplastic changes through a significant decrease in papillary projection and a reduced epithelial thickness **(**Fig. 2**/C)**.

### 3.4 Relaxation of the neck bladder smooth muscle strips

**Fig. 3:**
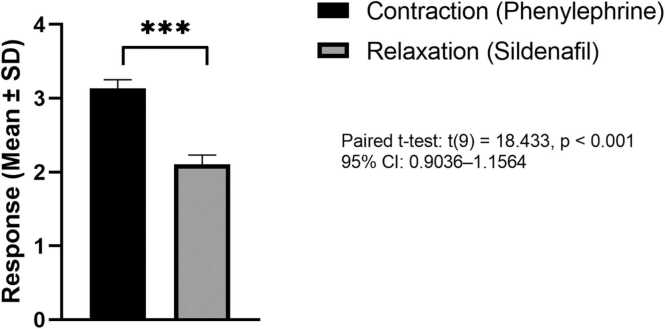
The relaxation effect of sildenafil on phenylephrine-induced contraction of bladder neck smooth muscle. The bar graph presents the responses (mean ± SD) of isolated bladder neck tissue strips (n = 10) to phenylephrine-induced contraction and subsequent relaxation after sildenafil administration. Sildenafil induced a significant relaxation response of the smooth muscle compared to phenylephrine alone. Asterisks (***) indicate statistically significant (p < 0.001), (paired *t*-test: t (9) = 18.433; 95 % CI: 0.9036–1.1564).

The relaxant effects of sildenafil on phenylephrine pre-contracted bladder neck smooth muscle strips were evaluated using organ bath experiments **(**Fig. 3**).** After stabilization, phenylephrine (10^-6 M) induced consistent contraction in all muscle strips (n = 10). Once a stable plateau was reached, cumulative concentration-relaxation curves for sildenafil were constructed.

Sildenafil induced a significant relaxation of the smooth muscle, reducing the mean contraction value from 3.14 ± 0.12 (±SD) to 2.11 ± 0.13 (±SD), representing a 32.8 % decrease in tension. The paired sample *t*-test in Table 1 and Fig. 3 revealed a mean difference of 1.03 ± 0.18 (±SD) between contraction and relaxation. This difference was statistically significant (p < 0.001).

**Table 1:** Paired *t*-test analysis of sildenafil-induced relaxation in the bladder neck smooth muscle.

Measure	Mean ± SD, (N)	95 % Confidence Interval of the Difference (Lower-Upper)	Mean Difference ± SD	t	df	P-value
Contraction (Phenylephrine)	3.14 ± 0.12 (N = 10)	0.90–1.16	1.03 ± 0.18	18.43	9	< 0.001
Relaxation (Sildenafil)	2.11 ± 0.13 (N = 10)					

**Fig. 4:**
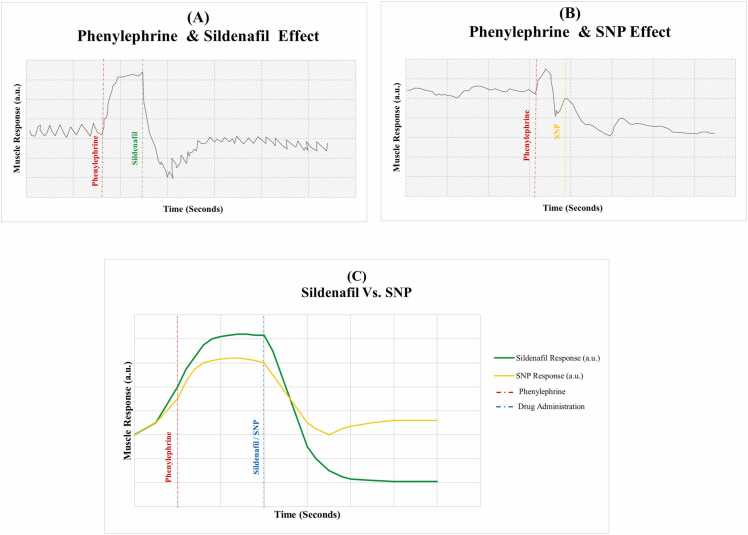
Effects of sildenafil and Sodium Nitroprusside (SNP) on Phenylephrine-Induced Contraction in Bladder Neck Smooth Muscle. **(A-B).** Organ bath tracings present the relaxant effects of sildenafil (Green Dashed Line, Fig. 4/A) and Sodium Nitroprusside (SNP) (Yellow Dashed Line, Fig. 4/B) on phenylephrine-induced contraction of bladder neck smooth muscle (Red Dashed Line, Fig. 4/A-B). The results demonstrate significant relaxation after adding sildenafil, confirming its ability to induce smooth muscle relaxation. **(C).** The overlay of tracings compared the relaxant effect of sildenafil (green line) and SNP (Yellow line) following Phenylephrine-Induced Contraction in Bladder Neck Smooth Muscle (Red Dashed Line). Drug administration points are indicated in the blue dashed line.

Additional pharmacological interventions were performed to evaluate if the sildenafil-induced relaxation was mediated through the NO pathway. The addition of L-Name (a NO synthase inhibitor) to the organ bath significantly inhibited the relaxation response, while Sodium Nitroprusside (SNP, an NO donor) enhanced it. Fig. 4**/A** presents the sildenafil-induced Relaxation; the red dashed line indicates phenylephrine-induced contraction of bladder neck smooth muscle strips. The green dashed line indicates a relaxation response following sildenafil administration, highlighting sildenafil's effect in reversing phenylephrine-induced contraction and promoting a sustained relaxation response. These results show that sildenafil induces a steep drop in the tracing and sustains stable smooth muscle relaxation. Fig. 4**/B** presents the phenylephrine-induced contraction and SNP-enhanced gradual relaxation, which is less deep and sustained compared to the sildenafil **(**Fig. 4**/A).** SNP, a nitric oxide donor, caused a decline in the tracing, inducing smooth muscle relaxation. Additionally, Fig. 4/C illustrates the overlay of tracings compared to the relaxant effect of sildenafil and SNP following Phenylephrine-Induced Contraction in Bladder Neck Smooth Muscle. The findings revealed that sildenafil-induced smooth muscle relaxation supports the role of NO in mediating smooth muscle relaxation. The ability of SNP to enhance relaxation directly by donating NO further supports the idea that sildenafil exerts a smooth muscle relaxation effect via NO-dependent mechanisms involved in NO-cGMP signaling pathways.

## 4 Discussion

This study examined the effect of sildenafil in counteracting the smooth muscle contraction of the bladder neck induced by phenylephrine and reversing testosterone-induced prostate hypertrophy. The results demonstrate the relaxant effects of sildenafil on the urinary bladder neck of a hypertrophic prostate. Histologically, our findings (Fig. 2A-C) revealed that testosterone-treated prostates (Fig. 2B) exhibited significant papillary projections, expanded acinar architecture, and increased epithelial thickness compared with the control (Fig. 2A) and Sildenafil-treated group (Fig. 2C), which are the hallmarks of prostate hyperplasia [2], [20]. Sildenafil treatment significantly reversed these structural changes, suggesting an influence on proliferative and inflammatory processes driving prostate growth (Fig. 2C).

The current study's results align with previous literature supporting the ability of PDE5Is to attenuate prostatic hyperplasia and enlargement. For example, Abubakar et al. (2024) demonstrated that sildenafil mitigated testosterone-induced hyperplastic alterations [21]. Çalışkan et al. (2017) [22] demonstrated that sildenafil, administered at a dose of 2 mg/kg in a testosterone-induced prostatic hyperplasia rat model, significantly reduced prostate weight and improved histopathological parameters, including epithelial thickness and stromal hyperplasia. Similarly, Mulhall et al. [23] demonstrated that PDE5Is reduced prostate weight and improved LUTS by inducing relaxation in the lower urinary tract tissues. The reduction in epithelial remodeling observed in this study (Fig. 2) confirms the findings shown by Kaplan et al. [24] and Vignozzi et al. [25]; the authors claimed that PDE5Is exhibited anti-inflammatory and antifibrotic effects on human myofibroblast prostatic cells via blunting Interleukin-8 (IL-8) and induced protein 10 (IP-10) secretion. PDE5Is also inhibit inflammation through the suppression of tissue remodeling marker gene expression by activating the cGMP/PKG signaling pathway [25].

A key aspect of this study is evaluating the relaxant effects of sildenafil on smooth muscle in the bladder and neck. The significant relaxation observed in phenylephrine-induced contractions, as illustrated in Fig. 3,highlights sildenafil’s potential to alleviate bladder obstruction and enhance urinary flow. This aligns with the proposed mechanism of PDE5Is, suggesting mediated smooth muscle relaxation via cGMP in response to NO [23], [24]. The dynamic tracing profiles in Fig. 4A–C demonstrate that sildenafil induces a smooth muscle relaxation pattern similar to that of sodium nitroprusside (SNP), supporting the involvement of the nitric oxide (NO)-cGMP pathway in mediating its relaxant effects. NO was initially identified as the "endothelium-derived relaxing factor" due to its ability to promote vascular relaxation; it is now known to regulate numerous functions, including those within the genitourinary system. Eryildirim et al. [26] demonstrated sildenafil’s positive impact on ED and LUTS by triggering the NO/cGMP cascades, which promote smooth muscle relaxation in the prostate and bladder in a dose-dependent manner.

The expression of PDE, especially in the transition zone of the prostate, supports the potential use of PDE5Is for treating prostate diseases [27]. Recent studies have shown phosphodiesterase type 5 (PDE5) mRNA expression in the rat urogenital tract, specifically in the bladder, urethra, and prostate. This suggests that medications targeting NO/cGMP pathways could be crucial in treating sub-vesical obstruction associated with lower urinary tract symptoms (LUTS) [13]. Preclinical and clinical studies have also shown that PDE5Is improve symptoms of BPH and LUTS, most likely due to their ability to relax smooth muscle via NO pathways and inhibit the proliferation of prostatic stromal cells [13], [26], [27]

Emerging evidence has also shown that treatment with PDE5Is not only enhances muscular wall relaxation but also may positively affect blood perfusion in the lower urinary tract, which restores function and morphologic changes in the bladder and prostate tissues [2], [25]. Bittencourt et al. [6] demonstrated that phenylephrine-induced bladder-neck relaxation was achieved with high sildenafil levels in constricted human bladders, suggesting that PDE inhibitors may potentially influence bladder relaxation. Grimsley et al. (2007) hypothesized that sildenafil improves ED and BPH/LUTS by relaxing the prostatic duct smooth muscle, enhancing the washout of prostatic reflux products [28]. Similarly, Mumtaz et al. [29] demonstrated that NO is an important inhibitory neurotransmitter in the smooth muscle of the urethra and increases the level of cGMP, which exerts a relaxant effect.

Although PDE5Is are generally considered safe drugs with few side effects [2] Long-term studies are needed to evaluate their impact on the typical male reproductive system, specifically on the prostate. The ultrastructural and histological analyses conducted in this study provide critical insights into the cellular and structural effects of sildenafil on prostate hyperplasia and relaxation. Our findings demonstrated that chronic treatment of rabbits with sildenafil 5 mg/kg/day for 8 weeks mitigates hyperplastic changes and preserves typical tissue architecture (Fig. 1). These results suggested that the chronic use of sildenafil does not cause evident prostatic damage and, therefore, seems safe for the treatment of prostatic disorders. However, reproductive safety, including its effects on sperm motility, morphology, and fertility, was not assessed in this study and should be investigated further in future studies to evaluate its reproductive safety.

## 5 Conclusion

This study reinforces the therapeutic benefits of PDE5Is in treating prostate hyperplasia and lower urinary tract symptoms (LUTS) by elucidating their antiproliferative and smooth muscle relaxant properties. These results contribute to the growing body of evidence supporting the integration of PDE5Is into the clinical management of prostatic disorders, paving the way for novel, non-invasive treatment strategies.

## CRediT authorship contribution statement

**Alzoubi Karem:** Writing – review & editing, Writing – original draft, Visualization, Supervision, Resources, Project administration, Investigation, Data curation, Conceptualization. **Qasim A. El-Dwairi:** Writing – review & editing, Writing – original draft, Visualization, Validation, Supervision, Methodology, Investigation, Funding acquisition, Data curation, Conceptualization. **Rania Mahafdeh:** Writing – review & editing, Writing – original draft, Validation, Supervision, Resources, Project administration, Investigation, Funding acquisition, Formal analysis, Data curation, Conceptualization.

## Authors’ Contributions

The authors confirm their contribution to the paper as follows: study conception and design: Qasim A. El-Dwairi, Karem H. Alzoubi, and Rania Mahafdeh; data collection: Qasim A. El-Dwairi; draft manuscript: Qasim A. El-Dwairi, Karem H. Alzoubi, and Rania Mahafdeh. Author. All authors reviewed the results and approved the final version of the manuscript.

## Consent for publication

Not applicable.

## Ethics approval and consent to participate

The study protocol was approved by the Institutional Animal Care and Use Committee (IACUC) at Jordan University of Science and Technology, Irbid, Jordan.

## Human and animal rights

The Institutional Animal Care and Use Committee at Jordan University of Science and Technology, Irbid, Jordan, approved the study protocol (Protocol number 20060134).

## Funding

A grant (No. 134-2006) from the Deanship of Research, Jordan University of Science and Technology, Irbid, Jordan, supported this project.

## Declaration of Competing Interest

The authors declare that they have no known competing financial interests or personal relationships that could have appeared to influence the work reported in this paper.

## Data Availability

Data will be made available on request.
